# Diagnostic Challenges for Clinicians in Lobular Breast Carcinoma With Peritoneal Carcinomatosis: A Case Report of an Immunocompromised Patient

**DOI:** 10.7759/cureus.65610

**Published:** 2024-07-28

**Authors:** Soufia El Ouardani, Hind Chibani, Meryem El Jarroudi, Mohamed Mouhoub, Ouissam Al Jarroudi, Hanane Hadj Kacem, Sami Aziz Brahmi, Said Afqir

**Affiliations:** 1 Medical Oncology, Mohammed VI University Hospital, Oujda, MAR; 2 Medical Oncology, Faculty of Medicine and Pharmacy of Oujda, Mohammed First University, Oujda, MAR; 3 Pathology, Mouhoub Pathological Anatomy Center, Oujda, MAR; 4 Radiology, Mohammed VI University Hospital, Oujda, MAR; 5 Radiology, Faculty of Medicine and Pharmacy of Oujda, Mohammed First University, Oujda, MAR

**Keywords:** diagnostic pitfall, pathology, tuberculosis, peritoneal carcinomatosis, invasive lobular breast carcinoma

## Abstract

Peritoneal carcinomatosis (PC) is a common condition in oncology. The lack of specificity in radiological and clinical characteristics of carcinomatosis makes their etiological diagnosis difficult. Metastatic infiltrating lobular breast cancer with PC is also a common occurrence in daily medical practice. We report the case of a 45-year-old female patient with chronic renal failure undergoing hemodialysis, admitted for lobular breast carcinoma with bone and peritoneal metastases. The surgical exploration including a biopsy revealed peritoneal tuberculosis. The focus of this paper is to discuss the diagnostic traps associated with PC in malignant tumors to highlight the importance of pathological evidence in such cases.

## Introduction

Breast cancer is the most common form of cancer in women [1]. Infiltrating lobular carcinoma (ILC) constitutes 5-15% of all breast cancers, and is the second most prevalent histological subtype of breast cancer following infiltrating ductal carcinoma (IDC) [2]. Metastatic ILC tends to spread to the gastrointestinal and peritoneal regions [3].

Peritoneal carcinomatosis (PC) is a common condition that often occurs in the progression of digestive and gynecological tumors [4]. The radiological and clinical examinations lack specificity to determine the origin of PC and hide traps for the inattentive clinician [5].

In this paper, we describe the case of an immunocompromised patient admitted for metastatic lobular carcinoma with carcinosis. After surgical exploration and biopsy, the patient has been diagnosed with peritoneal tuberculosis (PT). The diagnostic pitfalls of PC will be discussed.

## Case presentation

**Figure 1:**
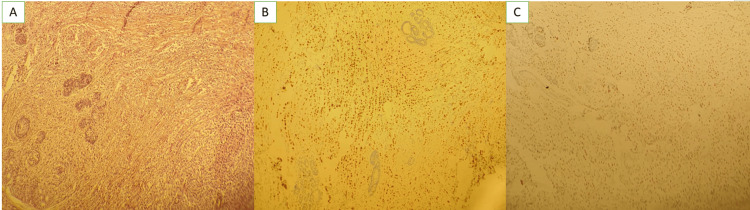
Photomicrographs of the breast biopsy (A) Mammary parenchyma extensively infiltrated by carcinomatous proliferation arranged in Indian files. Tumor cells show mild cytonuclear atypia (hematoxylin and eosin, magnification 10x). (B) Overexpression of estrogen receptors by tumor cells (diaminobenzidine, magnification 10x). (C) Overexpression of progesterone receptors by tumor cells (diaminobenzidine, magnification 10x).

A 45-year-old female patient undergoing chronic hemodialysis for three years with a history of hypertension. She was admitted to the oncology hospital for a lobular infiltrating carcinoma of the right breast with bone and peritoneal metastases. The patient had a good Performance Status (PS 1). The clinical examination of the right breast found an irregular, fixed, non-inflammatory mass measuring 5 cm and 2 homolateral axillary lymphadenopathies. The breast mass classified BI-RADS 5 (Breast Imaging Reporting and Data System) in mammography and ultrasound. A breast biopsy confirmed an ILC of Scarff-Bloom-Richardson (SBR) grade II (Figure 1A). The immunohistochemistry objectified a luminal A subtype (overexpression of estrogen receptors at 80% and progesterone receptors at 80%, human epidermal growth factor receptor 2 (HER2)-negative, and Ki67 at 10%) (Figures 1B-1C).

**Figure 2:**
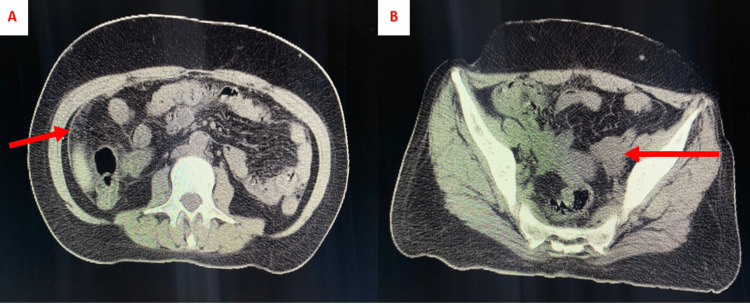
CT abdominopelvic sections of the patient (A) Thickening of the peritoneal sheets in favor of carcinomatosis (red arrow). (B) Enlarged left ovary with the presence of a solid-cystic mass of 3 cm (red arrow).

Cervical, thoracic, and abdominopelvic CT scans showed small nodules of carcinomatosis infiltrating the peritoneum, along with thickening and elevation of the peritoneal leaflets. The CT scan found also a solid-cystic left ovary mass of 3 cm, and the presence of axial and peripheral diffuse lytic bone metastases confirmed by a bone scan (Figure 2).

**Figure 3:**
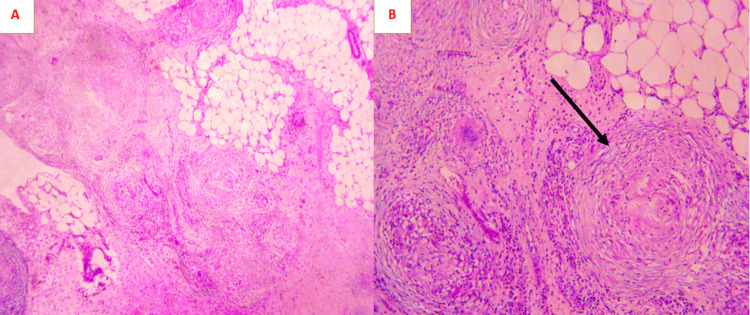
Photomicrograph of the peritoneal tuberculosis (A) Epiploic tissue is widely infiltrated by numerous epithelioid and gigantocellular granulomas, sometimes centered by caseous necrosis (hematoxylin and eosin, magnification 20x). (B) Epithelioid granuloma (black arrow) surrounded by a lymphocytic corona and centered by caseous necrosis (hematoxylin and eosin, magnification 40x).

**Figure 4:**
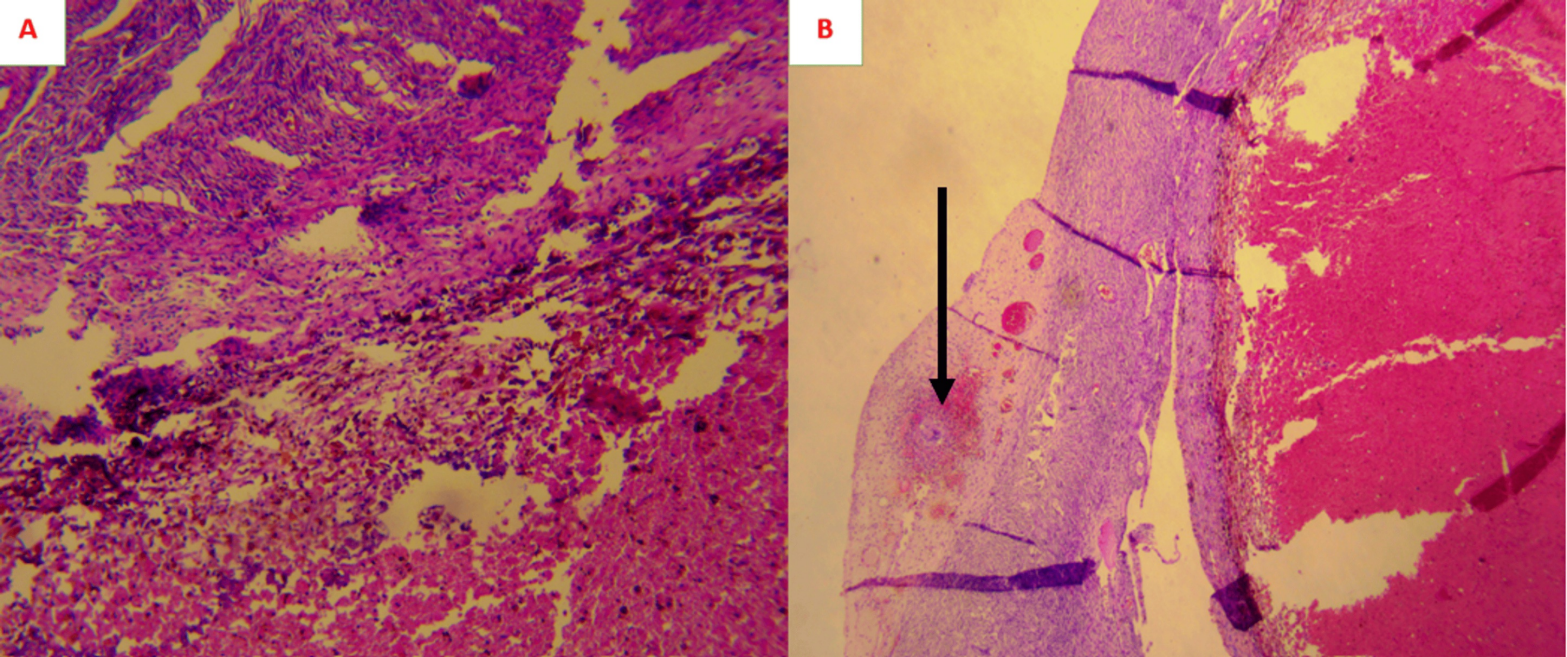
Photomicrograph of the ovarian cyst (A) Ovarian cyst lined with basophilic granular cells with regular nuclei in some areas and remodeled by hemorrhages and numerous siderophages (hematoxylin and eosin, magnification 20x). (B) Fibrous tissue adjacent to the ovarian cortex, the site of epithelioid and gigantocellular granulomas with focal caseous necrosis (black arrow) (hematoxylin and eosin, magnification 20x).

The multidisciplinary consultation meeting decided to perform an exploratory laparoscopy with ovary mass and carcinosis biopsies in order to rule out a synchronous primary ovarian cancer. Pathological examination of the epiploic biopsies showed caseous granulomas typical of tuberculosis (Figure 3), and the left adnexectomy revealed a benign follicular cyst with tuberculoid infiltration of the ovarian cortex (Figure 4).

Prior to initiating treatment with cyclin-dependent kinase 4 and 6 inhibitors (CDK 4/6 inhibitor) and aromatase inhibitors with castration as a first-line therapy for her metastatic lobular cancer (no visceral crisis), the patient received anti-tuberculosis treatment four weeks earlier with regular clinical and biological follow-up.

## Discussion

Breast cancer is the most prevalent form of cancer among women and the second most common cause of cancer-related deaths in women globally [1]. ILC is less common than IDC and accounts for 5-15% of breast cancers [2]. ILC differs from IDC in molecular, morphological, and clinical characteristics, which pose challenges in diagnosis and treatment [6]. The ILC is also known for its ability to spread to the gastrointestinal and peritoneal areas [3]. The occurrence of peritoneal metastases is uncommon with an estimated prevalence of 9.9% in ILCs compared to 2% in IDCs [6]. Peritoneal metastases are diagnosed using either biopsy following surgical examination, cytology of ascites fluid, or radiological criteria, as indicated in published research. At the molecular level, somatic loss of p53 and E-cadherin is associated with frequent peritoneal dissemination of ILC [7]. There is currently a lack of sufficient literature data to establish a standardized approach for caring for these patients and determining their survival rates [8]. Studies have tested intraperitoneal chemotherapy and cytoreduction surgery, and favorable outcomes have been observed in carefully selected patients with no residual disease after systemic treatment [7,9]. PC from breast cancer is associated with a bleak prognosis compared to other metastatic sites, due to the diagnostic challenge it poses and also its resistance to systemic treatment [10].

PC related to breast cancer may hide diagnostic traps for clinicians. The radiological and clinical characteristics of carcinosis lack specificity, making their etiological diagnosis difficult [5]. In the example of our patient, surgical examination and anatomopathological evidence revealed PT, which needs to be treated before starting systemic therapy for breast cancer.

Peritoneal lumps and nodules may not usually indicate PC [5]. Secondary tumor origin is more prevalent and typically occurs in the advanced stages of gastrointestinal and gynecological cancers [4]. However, it is important to note that other benign or malignant disorders, whether primary or secondary, may also be implicated [11]. Infections such as PT are uncommon and constitute a real diagnostic challenge, particularly in immunocompromised patients and in endemic countries like Morocco [12]. The diagnosis of PT is based on one or more of the following criteria: (1) pathological evidence showing the discovery of epithelioid granuloma with caseating necrosis, or the presence of other histological findings indicative of tuberculosis; (2) The identification of Koch's bacillus or acid-alcohol resistant (BAAR) through culture [13]. The conventional treatment involves the administration of anti-tuberculosis drugs for at least six months [12]. Actinomycosis is a bacterial infection caused by *Actinomyces* species, and the peritoneum is the third most commonly affected site [14]. Desmoid tumors, also known as mesenteric fibromatosis, is a benign condition and often idiopathic, although it can be linked to genetic diseases such as familial adenomatous polyposis [15]. Splenosis, endometriosis, and systemic diseases such as amyloidosis are also benign etiologies for carcinosis [11]. The clinician is required to assess the number of nodules, the location, the presence of another synchronous tumor, and the patient's history and must request histological proof in doubtful situations [16].

## Conclusions

PC in the context of tumor diseases poses many diagnostic challenges. Based on this presentation, it is important for the clinician to be attentive and provide histological evidence, particularly for patients with a compromised immune system, systemic diseases with a specific medical background, suggestive symptoms, and when there is suspicion of a concurrent tumor in the gastrointestinal or gynecological system.
